# Analysis of Molecular Markers of HPV Infection Persistence: A Narrative Review

**DOI:** 10.3390/cancers18060981

**Published:** 2026-03-18

**Authors:** Dominik Pruski, Sonja Millert-Kalińska, Katarzyna Wszołek, Victoria Musiałowicz, Jacek P. Grabowski, Robert Jach, Mustafa Zelal Muallem, Jalid Sehouli, Marcin Przybylski

**Affiliations:** 1 Department of Obstetrics and Gynecology, District Public Hospital in Poznan, 60-479 Poznan, Poland; millertsonja@gmail.com (S.M.-K.);; 2 Department of Maternal and Child Health, Poznan University of Medical Sciences, 60-806 Poznan, Poland; 3 Department of Gynecology with Center for Oncological Surgery, Charité-University Medicine of Berlin, Campus Virchow Klinikum, 13353 Berlin, Germany; 4 Division of Gynecologic Endocrinology, Jagiellonian University Medical College, 31-501 Krakow, Poland

**Keywords:** HPV infection persistence, narrative review, HPV, HPV DNA testing, HPV mRNA testing, HPV DNA methylation test

## Abstract

Background: Persistent infection with high-risk human papillomavirus (hr-HPV) is the necessary agent of cervical cancer, yet its molecular definition remains heterogeneous. Multiple molecular approaches have been developed to characterize HPV persistence, including repeated detection of viral DNA, assessment of viral oncogene expression, and analysis of HPV-related DNA methylation. These approaches originate from different scientific traditions and reflect distinct conceptualizations of persistence. Objective: To synthesize and compare molecular methods used to detect persistent HPV infection through a narrative review and to clarify how different biomarkers conceptualize HPV persistence and disease progression. Methods: We conducted a narrative review in accordance with the RAMESES guidelines. Medline, Scopus, and the Cochrane Library were searched for original studies published between 2016 and 2025 investigating molecular markers of HPV persistence. An interpretive synthesis was performed to identify research traditions, underlying assumptions, and clinical implications. Results: Three major molecular narratives were identified. Persistent DNA positivity defines persistence as repeated detection of the same HR-HPV genotype over time and reflects an epidemiological–virological perspective with high sensitivity but limited specificity. Persistent oncogene expression, assessed by E6/E7 mRNA detection, conceptualizes persistence as active viral oncogenic activity and shows improved specificity for clinically relevant lesions. Persistent epigenetic imprint, measured by DNA methylation of viral and host genes, captures cumulative biological effects of long-term infection and is strongly associated with high-grade lesions and cervical cancer. These narratives represent complementary stages along a continuum of molecular persistence. Conclusions: Molecular markers of HPV persistence reflect the evolving understanding of cervical carcinogenesis, progressing from repeated viral DNA detection to oncogenic activity and stable epigenetic alterations. These complementary biomarkers represent different biological stages of persistent infection and may improve risk stratification in HPV-based screening and triage strategies.

## Simple Summary

Persistent infection with high-risk human papillomavirus is the key driver of cervical cancer development. Molecular diagnostics have evolved through three complementary approaches that reflect different biological stages of HPV persistence. The earliest approach, HPV DNA testing, identifies repeated detection of the same viral genotype over time and provides high sensitivity for identifying infection but limited specificity for predicting disease progression. The second approach, HPV E6/E7 mRNA testing, detects expression of viral oncogenes responsible for cell cycle dysregulation, representing active oncogenic infection and offering improved specificity for clinically relevant lesions. The most recent approach involves DNA methylation biomarkers, which measure epigenetic alterations in viral and host genes associated with long-term infection, viral integration, and progression to high-grade cervical lesions or cancer. Together, these molecular markers represent a continuum from viral presence to oncogenic activity and stable cellular transformation, providing complementary tools for improved risk stratification and more precise cervical cancer screening and triage strategies.

## 1. Introduction

Human Papillomavirus (HPV) is the most common sexually transmitted infection in the human population. According to World Health Organization reports, in 2024, cervical cancer was diagnosed in 670,000 cases and was the cause of 350,000 deaths worldwide. This makes it the fourth most frequent cancer in women globally. Therefore, the WHO announced in May 2018 a global strategy to eliminate cervical cancer by 2030, following the 90–70–90 targets. Ninety percent of girls should be fully vaccinated by age 15, 70% of women have access to high-performance screening tests, and 90% of women with precancerous lesions and diagnosed invasive cancer cases receive appropriate treatment [1]. HPV infection prevalence peaks in two age groups. Young adults aged 18–25 years are the most sexually active group. The second peak occurs around age 50, when entering new relationships. Around 90% of infections are transient, meaning they are cleared spontaneously within 2 years of primary detection. The remaining 10% remains persistent, eventually leading to invasive cancer if the neoplasia remains untreated [2]. Several factors have been identified that can contribute to the persistence of HPV infection: having many pregnancies, a high number of sexual partners, genetic predispositions, smoking, the use of immunosuppressive medications, and co-infections with other sexually transmitted pathogens such as herpes simplex virus type 2 and Chlamydia trachomatis [3,4].

Ongoing research is examining the role of the vaginal microbiome in the development of precancerous and cancerous lesions. Alterations in the microbiome may impair viral clearance, thereby promoting persistent infection [5,6]. More than two hundred viral genotypes have been identified. The vast majority of cervical cancers are associated with persistent infection by high-risk types, including 16, 18, 31, 33, 35, 39, 45, 51, 52, 56, 58, and 59. A breakthrough in the diagnosis of precancerous lesions and cervical cancer was Harald zur Hausen’s discovery. The scientist proved that human papillomavirus infection is responsible for carcinogenesis, and this way is recognized as the principal factor in the development of cervical cancer. Persistent HPV infection may lead to the development not only of cervical cancer, but also of anogenital (vulvar, vagina, penile, anal) and oropharyngeal cancers. Apart from malignant lesions, benign papillomas may occur in the anogenital area as well as in the oral cavity [7].

**Figure 1:**
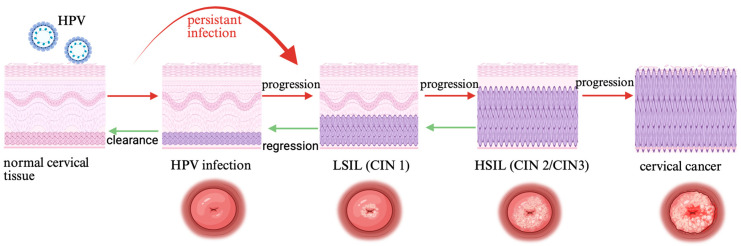
The natural process of HPV infection in the cervical epithelium. HPV—human papillomavirus, CIN—cervical intraepithelial neoplasia, LSIL—low-grade squamous intraepithelial lesion, HSIL—high-grade squamous intraepithelial lesion.

Detection methods for precancerous conditions and cervical cancer have evolved over the years, from the conventional Pap smear, through liquid-based cytology, to the link with the human papillomavirus and molecular assessment of HPV infection in the cervix. Subsequent molecular approaches include HPV mRNA testing, methylation testing, immunocytochemical assessment of p16 and Ki-67 in cytological specimens, and immunohistochemical detection of E4, E6, and E7 proteins in cervical tissue specimens. Cutting-edge medical discoveries are supporting physicians, including the use of AI in colposcopic software, devices such as DYSiS, which uses advanced photonics to measure the speed, intensity, and duration of the acetowhitening process on the cervix, creating a special map of the cervix with suggested biopsy areas. Some innovative methods, but with little clinical application so far, are spectroscopic methods—LuViva, and optoelectronic methods—Truscreen and Zedscan. The field of cervical cancer screening has evolved across multiple research traditions, each with distinct conceptual foundations, theoretical parameters, and methodological assumptions. Because these traditions have developed in parallel and often produce incompatible findings, a narrative review—designed to map and compare storylines of scientific inquiry—provides a suitable approach. Our work focuses in particular on the historical approach to molecular markers detecting features of persistent HPV infection—HPV DNA positivity, the presence of HPV mRNA, and the detection of HPV DNA methylation. The natural history of HPV infection in the cervical epithelium and the risk of progression and regression are shown in Figure 1.

### 1.1. What Is Already Known on This Topic?

- Cervical cancer is primarily caused by persistent infection with high-risk human papillomavirus (HPV).
- Conventional cytology (cytology) has historically reduced mortality from cervical cancer but has limited sensitivity and reproducibility.
- HR HPV testing is increasingly recommended as the primary screening method worldwide.
- Molecular markers (HPV mRNA, HPV DNA genotyping, DNA methylation) are considered indicators of persistent HPV infection and are actively assessed for triage and subsequent treatment decisions.

### 1.2. What This Paper Adds

- This review synthesizes the evolution of diagnostics for persistent HPV infection in the cervix across three research traditions: detection of HR HPV in serial assays, HPV mRNA testing, and assessment of HPV DNA methylation in the cervix.
- It presents a perspective on future diagnostics, highlighting molecular markers for screening strategies in the era of diagnostic tragedies and the search for the most convenient methods for patients.
- Using a narrative approach, the article integrates diverse scientific paradigms into a coherent account of “yesterday, today, and tomorrow” in the diagnosis of persistent HR HPV infection leading to cervical cancer.

### 1.3. Aim of the Narrative Review

This study aims to present a narrative review to synthesize available evidence on differences in approaches to molecular diagnostic methods for persistent HPV infection.

### 1.4. The Specific Review Questions Are

1. What studies (or epistemic traditions) have addressed inequalities in molecular methods for detecting persistent HPV infection in the cervix?
2. How has each tradition conceptualized this topic, and what methods have they used?
3. What theoretical assumptions about how and why inequalities in methods for detecting persistent HPV infection in the cervix persist are present in these narratives?
4. What changes have been observed in the narratives, and what has prompted these changes?
5. What conclusions can be drawn by combining and comparing the results of different traditions?
6. How does a narrative approach improve understanding of inequalities in molecular methods for detecting persistent HPV infection in the cervix?

### 1.5. Eligibility Criteria

Studies were included if they met the following criteria:

1. Reported original research,
2. Investigated molecular markers used to define or assess persistent hrHPV infection in the cervix,
3. Addressed HPV DNA detection, HPV oncogene (E6/E7) mRNA expression, or HPV-related DNA methylation,
4. Were published in English.

Review articles, analyses, commentaries, discussion papers, conference abstracts, and studies not focused on cervical HPV infection were excluded.

## 2. Methods

**Figure 2:**
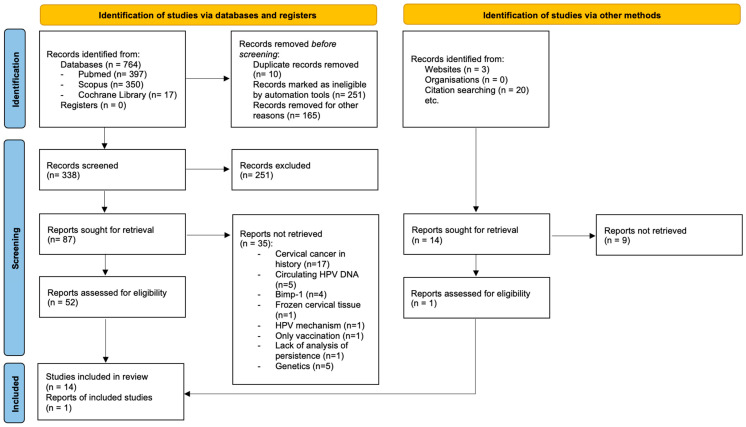
PRISMA scheme modified for a narrative review.

The narrative review was methodologically designed using the RAMSES [8] and Greenhalgh et al.’s [9] methodological guidance for planning, searching, mapping, appraisal, and synthesis. The PRISMA guidelines have been included for complete transparency [10]. PRISMA scheme modified for a narrative review is shown in Figure 2. Three databases (Medline, SCOPUS, and Cochrane Library) were searched for full-text articles published between 2016 and 2025 related to molecular methods for detecting persistent HPV infection in the cervix. Literature reviews, meta-analyses, and discussion papers were excluded. Keywords used during search were as follows: (marker* OR biomarker*) AND (persistent OR persistence OR persist*) AND (cervical) AND (HPV OR “human papillomavirus”). The registration number at the International Prospective Register of Systematic Reviews is 1291648.

Narrative reviews are a growing, increasingly important approach to qualitative and mixed-methods systematic reviews that enable the synthesis of heterogeneous information drawn from different paradigms [8,9]. The six guiding principles of the narrative review—Pragmatism, Pluralism, Historicity, Contestation, Reflexivity, and Peer review—are integrated into the review process as articulated in Table 1. Given the development of diagnostic and detection methods for precancerous conditions and cervical cancer, a narrative review approach will enable researchers to identify, formulate, synthesize, and interpret the diverse literature on various approaches to molecular markers of persistent HPV infection, which is the initial stage of cervical cancer development.

### 2.1. Selection and Appraisal

Titles and abstracts were screened, and relevant articles were assessed in full by the lead investigator (DP) for eligibility using the technique of survey, question, read, recall, and review. Outcomes were peer-reviewed by project supervisors (JG and RJ), and uncertainties were discussed for consensus. Research articles focusing on molecular markers of HPV persistence were included. Literature reviews, concept analyses, commentaries, and discussion papers were excluded. We also excluded text in languages other than English.

### 2.2. Data Extraction

Details of included articles (author, year, title, journal, country) and their abstracts (aim, methods, sample, key findings, conclusion) were extracted and compiled into a summary table for this review.

### 2.3. Data Analysis and Synthesis

Data analysis and synthesis involved an interpretive process of immersion in the data and regular discussions between the research team to consider how the data addressed the research aim, the synthesis of concepts, and the development of narratives [8]. Frequency counts were obtained to describe characteristics of included articles and capture the development of the research topic over time. Interventions and key findings were analysed thematically using an adapted reflexive approach through familiarisation with the data, generating categories, constructing and revising sub-themes, defining themes, and presenting results. Background information was analysed to identify the historical basis for the research (principle of historicity), that is, the research purpose, key molecular markers of HPV infection persistence concepts or assumptions that underpinned the research, and commonly cited authors or works. Research purpose data were categorised into common themes that emerged from the literature.

## 3. Results

### 3.1. HPV Genotyping Test

Following Professor Harald zur Hausen’s discovery, numerous investigators focused on detecting human papillomavirus (HPV) DNA in cervical tissue specimens. In 1988, the U.S. Food and Drug Administration (FDA) approved the first commercially available assay for detecting HPV genetic material. In the year 1983, HPV-16, the most oncogenic and prevalent type, was first identified in cervical cancer tissue. Next year, another high-risk HPV-18 genotype was identified. After years of research by Professor Harald zur Hausen on the HPV virus, infection with the highly oncogenic HPV was linked to an increased risk of precancerous conditions. This led to the awarding of the Nobel Prize in Medicine to Professor Hausen’s team in 2008. This prompted research into the application of HPV detection in the cervix in clinical practice.

At the turn of the 1990s and 2000s, the first reports linking persistent HPV infection with the risk of advanced precancerous conditions of the cervix, such as CIN 2+, began to appear. Ho GY et al. were among the first to present results from a cohort study of young women in 1995, showing that most HPV infections are transient. Researchers proved that persistent HPV infections increase the risk of precancer lesions and cervical cancer [10]. The results of another study published in the same year confirmed that the phenomenon of persistent infection with the same HPV genotype carries an increased risk of progression to cervical intraepithelial neoplasia (CIN) [11]. In parallel, HPV genotypes 6 and 11 were also discovered, which were associated with changes that did not cause cervical cancer [12]. Thus, the first division into “oncogenic” and “benign” genotypes was created. In the following years, new HPV genotypes were discovered and sporadically detected in cancers (e.g., HPV 31, 33, 35). Only the results of cohort studies introduced a new category of potentially oncogenic genotypes. The first official classification of genotypes was introduced in 2003 by the International Agency for Research on Cancer, which divided genotypes into Group 1 (oncogenic), Group 2A/2B (probably/potentially carcinogenic), and Group 3 (non-oncogenic) [13]. The final tripartite division of HPV genotypes was published in 2003 in the New England Journal of Medicine, where the classification into oncogenic, potentially oncogenic, and non-oncogenic types was clear [14]. For nearly a quarter of a century, we have been accompanied by the following:

- high-risk: 16, 18, 31, 33, 35, 39, 45, 51, 52, 56, 58, 59,
- probable high-risk: 26, 53, 66, 68, 73, 82,
- low-risk: 6, 11, 40, 42, 43, 44, 54, 61, 70, 72, 81.

With advances in technology, the Polymerase Chain Reaction (PCR) has been used to detect human papillomavirus. PCR uses fluorescent probes that bind to the amplified DNA, enabling the real-time quantification and detection of specific HPV types. A variant of this method is multiplex PCR, which amplifies multiple HPV types in a single reaction for simultaneous detection and genotyping. The advantages of this method may include high sensitivity and specificity, rapidity, the ability to quantify viral load, and the potential to identify specific viral types (genotyping). On the other hand, high sensitivity can lead to false positives from contamination; some methods are technically complex or costly.

Currently, the two most popular methods are available: the Linear Array test, which uses PCR and hybridization, and the Cobas test, which uses real-time PCR [15,16]. The Linear Array test can detect multiple HPV genotypes with high accuracy, unlike the Cobas method, which detects only a limited number of types. They also differ in the reading method—strips versus digital smears—and the degree of test automation.

Initially, HPV DNA testing was recommended exclusively for women with abnormal cervical cytology categorized as atypical squamous cells of undetermined significance (ASC-US) as part of follow-up, to identify those who would benefit from referral for colposcopic examination. Despite the breakthrough in the diagnosis of cervical precancerous lesions represented by the introduction of HPV DNA testing, researchers encountered a significant limitation: high-risk HPV DNA tests do not directly detect cervical neoplasia but rather indicate an increased risk of its development. These assays do not differentiate between transient and persistent HPV infections. Consequently, HPV DNA testing demonstrates high sensitivity but limited specificity.

Most HPV infections (approximately 80–90%) are transient and do not lead to the development of precancerous conditions or cervical cancer. This typically affects young women, and the infection resolves within 6–24 months. In this case, HPV DNA does not integrate into the host genome. Much less frequently, HPV infection is persistent and leads to the development of SIL. If the same highly oncogenic HPV type persists in the cervix for 12–24 months, it might lead to integration of HPV DNA into the host cell genome.

### 3.2. HPV mRNA Test

The implementation of screening programs and new molecular techniques for assessing the presence of HPV infection in cervical tissues contributed to a decline in the number of women developing cervical cancer. Similar trends were observed in cervical cancer deaths in the United States and European countries after the 1990s and in the following decade. However, it became necessary to distinguish between incident and persistent infection. While HPV DNA tests were the standard by 2007, with DNA tests like HC2 (Digene, Gaithersburg, MD, USA) approved for co-testing in 2003, the first mRNA HPV tests, like the APTIMA (Gen-Probe, San Diego, CA, USA), and the PreTect HPV-Proofer (detecting E6/E7 mRNA for types 16, 18, etc.) emerged a bit later, used as triage tools, with significant adoption and studies in the late 2000s [17,18,19,20]. These tests began to lead the mRNA protocols by around 2010–2011, offering better specificity than DNA tests. In 2007, mRNA tests and the precise E6/E7 extension were used as molecular markers of HPV oncogenic activity. These tests demonstrated higher specificity than general HPV DNA testing [21]. Currently, there are three most common commercial tests for detecting hrHPV E6/E7 messenger RNA (mRNA). The PreTect HPV-Proofer (NorChip AS, Klokkarstua, Norway) and the NucliSENS Easy Q HPV (bioMérieux, Marcy-l’Étoile, France) are based on the same technology. Still, they are produced by different companies, with small differences in mRNA extraction protocol and data analysis [22,23]. The third one—the APTIMA HPV Assay (Hologic, San Diego, CA, USA)—is a target-amplification nucleic acid probe test for the qualitative detection of E6/E7 viral mRNA [24]. All tests received the CE-IVD mark, and the APTIMA HPV assay is additionally an FDA-approved test. The HPV mRNA tests differ not only in their biological principle but also in the range of genotypes they detect—from a broad, pooled panel of 14 HR-HPV (Aptima, Woburn, MA, USA) to selective detection of the five most oncogenic types—16, 18, 31, 33, 45 (PreTect, NucliSENS), reflecting different strategies for conceptualizing persistent infection.

The advantage of using HPV mRNA tests is their similar sensitivity combined with slightly higher specificity compared to HPV DNA tests for the detection of CIN 2+ lesions [25]. Furthermore, HPV mRNA tests can also be used for follow-up after treatment for CIN lesions (test-of-cure). Limitations of this method include a limited number of detectable genotypes and the fact that they cannot be used combined with self-sampling for cervical cancer screening.

### 3.3. Dual-Staining p16/Ki67

A separate issue is the dual-staining p16/Ki67, which has recently been proposed as a new triage tool for HPV infections instead of LBC in a population of HPV-positive women [26,27,28]. In 2020, p16/Ki67 DS was approved by the Food and Drug Administration for the triage of HPV-positive women to colposcopy. Many studies have investigated and validated DS’s high detection rate of CIN compared to cytology or co-testing. This relatively novel approach may also have other uses in the cervical cancer screening and management pathway.

### 3.4. HPV DNA Methylation Test

The late 20th century also saw the development and understanding of epigenetics’ role in cancer. Epigenetic changes (such as DNA methylation or histone modifications) do not alter the DNA sequence, but by influencing gene expression, they play a key role in cancer development. Epigenetic changes can result in the deactivation of tumor suppressor genes (inhibiting growth) or the activation of oncogenes (promoting growth), leading to uncontrolled cell proliferation. The first reports of HPV genome methylation appeared in 1987, when it was observed that HPV DNA integrated into the host genome exhibited epigenetic changes (including methylation). Researchers from Germany published a paper, “The effect of DNA methylation on gene regulation of human papillomaviruses” in 1993 [24]. These data were groundbreaking and suggested that DNA methylation is an important regulatory pathway in modulating HPV expression and, consequently, the proliferation rate of virus-infected cells. Methylation of the HPV genome and host genes is a key epigenetic mechanism associated with long-term infection persistence, viral integration, and progression to CIN3+ and cervical cancer. High methylation of the L1/L2 regions and the *CADM1*, *FAM19A4*, and *miR124-2* genes is characteristic of transforming infections and has significant diagnostic and prognostic implications.

HPV genome methylation affects cytosines within CpGs in viral DNA and increases with the duration of infection, HPV integration into the host genome, and progression of epithelial lesions. Methylation of the L1/L2 regions (so-called late genes) is low in transient infection, whereas methylation is high in CIN 2+ lesions and cervical cancer. Methylation of these regions correlates with the loss of the productive viral cycle and indicates the transition from the replicative to the transforming phase. Clinically, L1/L2 methylation is one of the best markers of cervical lesion progression. The regulatory gene E2 inhibits the expression of the E6/E7 oncogenes, and E2 methylation leads to loss of E2 control over E6/E7, promoting neoplastic transformation. Regarding the host genome, chronic HPV infection disrupts host cell epigenetics and hypermethylates tumor suppressor gene promoters. This leads to their silencing, loss of proliferation control, and epithelial destabilization. CADM1/FAM19A4/miR124-2 methylation is a molecular biomarker strongly correlated with CIN2+ lesions and progression to cervical cancer and is also used in the triage of HPV-positive patients.

Commercial HPV methylation tests, such as GynTect^®^, QIAsure Methylation Test, and CisCer^®^, measure methylation at specific loci in the viral and host genomes to identify hrHPV-positive women at high risk of CIN2+ or CIN3+ lesions. Unlike DNA and mRNA tests, these panels analyze persistent epigenetic “footprints” of long-term infection, offering high specificity for triaging HPV-positive women.

All identified and analysed studies are described in Table 2.

## 4. Discussion

The present narrative review demonstrates that the concept of persistent high-risk HPV infection has been shaped by three distinct molecular narratives, each emerging from different scientific traditions and addressing different dimensions of viral persistence. These narratives—persistent DNA positivity, persistent oncogene expression, and persistent epigenetic imprint—reflect an evolving understanding of HPV biology and carcinogenesis, progressing from viral presence to functional activity to long-term cellular reprogramming.

The earliest and most widely implemented narrative, persistent DNA positivity, conceptualizes persistence as the continued detection of HPV DNA during subsequent cervical swabs. This approach, rooted in epidemiology and virology, was instrumental in establishing HPV as the necessary causal factor of cervical cancer and in transforming screening strategies worldwide. Longitudinal cohort studies demonstrated that repeated detection of the same high-risk genotype confers a substantially increased risk of CIN2+ lesions and cervical cancer. However, despite its high analytical sensitivity, HPV DNA testing cannot distinguish between transient and clinically meaningful infections. As a result, this narrative inherently prioritizes sensitivity over specificity, leading to over-referral and the need for additional triage strategies.

In response to these limitations, a second narrative emerged, defining persistence as persistent oncogene expression, measured by detection of E6/E7 mRNA. This paradigm represents a conceptual shift from viral presence to viral activity. By focusing on transcriptional activity directly responsible for cell cycle deregulation and malignant transformation, mRNA-based assays better align with the biological mechanisms underlying disease progression. Consequently, HPV mRNA testing offers higher clinical specificity for CIN2+ lesions. Nevertheless, this approach captures only infections with ongoing oncogenic transcription and may fail to identify lesions in which viral activity is intermittent or has already induced downstream cellular alterations.

The most recent narrative, persistent epigenetic imprint, extends the concept of persistence beyond real-time viral detection to encompass the cumulative biological consequences of long-term infection. DNA methylation of viral regions (L1/L2, E2) and host tumor suppressor genes (e.g., *CADM1*, *FAM19A4*, *miR124-2*) reflects sustained exposure to oncogenic stimuli, viral integration, and transition from productive to transforming infection. Importantly, methylation-based markers are less dependent on current viral transcription and instead capture a stable molecular footprint of past and ongoing oncogenic processes. This characteristic explains their strong association with CIN3+ and invasive cancer and their high specificity in triage of HPV-positive women.

These three narratives should not be interpreted as competing diagnostic strategies but rather as complementary perspectives along a continuum of molecular persistence. HPV DNA positivity identifies viral presence, HPV mRNA expression indicates active oncogenic interference, and DNA methylation marks the accumulated epigenetic consequences of prolonged infection. This integrative framework clarifies why some HPV DNA–positive but mRNA–negative infections may still progress and why methylation markers can predict progression even in the absence of detectable viral transcription.

In conclusion, the narrative approach reveals that advances in HPV diagnostics mirror a broader conceptual evolution—from detecting the virus, to understanding its activity, to recognizing its lasting molecular legacy. Future screening and triage strategies are likely to benefit most from integrative models that combine these narratives, rather than privileging any single biomarker in isolation.

## 5. Conclusions

Molecular markers of HPV persistence reflect an evolving understanding of cervical carcinogenesis, progressing from viral presence to functional activity and stable epigenetic reprogramming. Integrating these complementary approaches may improve risk stratification and future HPV-based screening strategies. In particular, combining viral detection methods with host epigenetic biomarkers may allow for more precise identification of infections with true oncogenic potential. Further prospective studies and clinical validation are required to determine the optimal integration of these markers into routine cervical cancer screening and triage algorithms.

## Figures and Tables

**Table 1:** Six guiding principles of narrative reviews.

Principle	Definition	Application in This Review
Pragmatism	The included information should be driven by usefulness to the intended audience.	This review aims to understand the main paradigms or epistemic traditions that have sought to explain the differences in molecular markers of persistent HPV infection. In a diverse field of research and practice, articulating the complementary and conflicting approaches to understanding the problem across multiple disciplines is critical to attaining coherence and achieving convergent recommendations for patients.
Pluralism	The topic should be considered from multiple perspectives.	We will analyze current data on various approaches, persistent HPV HR DNA, HPV mRNA presence, and HPV DNA methylation.
Historicity	The included information should be presented in the order of its development over time.	The history/genealogy of the different epistemic traditions will be analysed using bibliometric methods. Landmark documents will be recorded and traced to study the evolution of the paradigms.
Contestation	Any conflicting information should be used to generate higher-order insights.	The differences between assessing molecular markers of persistent HPV infection and the recommended further diagnostic and therapeutic procedures will be highlighted to establish consensus on theories, methods, and approaches to the problem.
Reflexivity	There should be continual reflection on the review findings.	The narrative review will be updated to reflect the changes to the process as findings emerge.
Peer review	The review findings should be presented to an external audience for feedback.	The research results will be presented to scientists, gynecologists, and patients, as well as during a scientific conference.

**Table 2:** Description of studies.

Study	Molecular Biomarker	Timeframe	Objectives	Methods	Results	Conclusions	Definition
Bruno MT et al. [29]	NNO-LiPA HPV Genotyping Extra II assay (Fujirebio Inc., Tokyo, Japan)	2016–2021	To evaluate the clinical evolution of CIN2 in an HPV-positive cohort followed under active surveillance for up to 48 months, with particular attention to the risks of progression, persistence, and regression, and to associated predictors.	Retrospective observational study conducted on a cohort of 237 HPV-positive women with a histological diagnosis of CIN2. Patients underwent follow-up, including cytology and colposcopy every 6 months, and HPV testing annually.	After a maximum follow-up of 48 months, 61.3% of lesions regressed spontaneously, 13.1% persisted, and 25.5% progressed to CIN3. In multivariate analysis, high-grade cytology (ASC-H/HSIL) and HPV 16/18 infection were independently associated with a reduced likelihood of regression (*p* < 0.01) and an increased risk of progression (*p* < 0.05).	The combination of high-grade cytology and HPV 16/18 represents a high oncological risk profile, for which prompt treatment is indicated. Persistence beyond 36 months should be considered a cumulative risk marker, with implications for personalized follow-up and risk management.	Persistence: unchanged CIN2 diagnosis at sequential histologic evaluation beyond 12 months. Progression: histologic confirmation of CIN3 or adenocarcinoma in situ (AIS) at any point during follow-up.
Dübbel, L et al. [30]	methylation marker (*ZNF671*) from the complete GynTect^®^ kit	2016–2022	To study the main marker (*ZNF671*) methylation in formalin-fixed, paraffin-embedded (FFPE) material to determine whether the kit provides prognostic information as well.	289 FFPE samples from 139 patients were tested with varying CIN grades and disease trends, including regressive, persistent, progressive, and recurrent disease. Additionally, age and human papillomavirus (HPV) status were correlated with the results.	Although there are differences between FFPE material and cervical scrapes, a similar increase in *ZNF671* methylation with increasing neoplasia grade was observed (dysplasia-free: 0%, CIN 1: 8.20%, CIN 2: 26.73%, CIN 3: 32.43%, carcinoma: 100%). *ZNF671* is more likely to detect recurring (27.12% of positives) and progressive (59.32% of positives) neoplasia. Patients with HPV 16 (+) and >30 years are more likely to have *ZNF671* methylation-positive. However, patients < 30 years with persistent neoplasia also show methylation more frequently.	The methylation of *ZNF671* is measurable in cervical FFPE material and has prognostic value. Since *ZNF671*-methylated samples are most likely to be progressing, we recommend closer monitoring of patients with GynTect^®^-positive test results.	Persistent: patients showed stable biopsy-confirmed CIN 1 or CIN 2 without treatment. Progressive: patients showed a worsening disease trend; these patients were treated via conization for CIN 2 or CIN 3 grade.
Huang et al. [31]	HPV DNA test (Cobas^®^ 4800 Test, Roche Molecular Systems, Alameda, CA, USA)	August 2020–September 2021	To investigate whether persistent human papillomavirus integration at the same loci (PHISL) before and after treatment can predict recurrent/residual disease in women with CIN2–3, and to contrast it with the other predictors of residual or recurrent disease following CIN2-3 therapy	It is an observational cohort study that included 151 consecutive women diagnosed with CIN2-3 lesions who underwent LEEP or CKC. All HPV-positive cases were prospectively tested for HPV integration (via whole-genome sequencing and high-throughput viral integration detection) approximately 6 months after conization.	56 were HPV integration-positive, and 95 had HPV integration-negative results. 10.7% of HPV integration-positive patients had recurrence. Among the seven patients who tested with PHISL, six (85.7%) had residual/recurrent disease. PHISL was a prominent predictor of persistent/recurrent disease. The HPV test, the HPV integration test, and PHISL all had a sensitivity of 100% and a NPV of 100% for residual/recurrent disease. PHISL showed better specificity (98.0% vs. 82.0%, *p* = 0.005) and PPV (85.7% vs. 40.0%, *p* = 0.001) than the HPV test for predicting recurrence.	The HPV-integration-positive CIN2-3 women had much higher relapse rates than HPV-integration-negative CIN2-3 women. The findings indicate that PHISL derived from preoperative and postoperative HPV integration tests may be a precise biomarker for identifying residual/recurrent CIN 2/3.	PHISL-persistent human papillomavirus integration at the same loci
Damgaard RK et al. [32]	HPV genotyping was performed on archived tissue samples using the HPV SPF10-DEIA-LiPA25 system (DNA ELISA HPV SPF10 kit and RHA HPV SPF10-LiPA25 kit).	2000–2010	This study aimed to describe human papillomavirus HPV type-specific persistence/progression in women undergoing active surveillance for cervical intraepithelial neoplasia grade 2.	Women were identified from the Danish Pathology Data Bank (DPDB) and were considered to be undergoing active surveillance if they had a first cervical biopsy within 2 years of the index diagnosis and no loop electrosurgical excision procedure before this.	52.2% of all women had CIN ≥ 2 during follow-up; 70.5% were HPV-16 (+) and 29.5% were positive for other HPV types. HPV16 was associated with a significantly higher risk of persistence/progression compared with non-human papillomavirus-16. The risk of persistence/progression was highest in HPV-16 (+) women with a high-grade index cytology compared with HPV-16 (+) women with a low-grade cytology.	The highest risk of persistence/progression was observed among HPV-16 (+) women, particularly those with associated high-grade cytology. These findings suggest that early excisional treatment should be considered in this group of women.	Persistence/progression was defined as having a record of cervical intraepithelial neoplasia grade ≥ 2 in the DPDB determined on the last and worst diagnosis on a biopsy or loop electrosurgical excision procedure specimen during follow-up.
Bruno, M.T. et al. [33]	MAG NucliSenseasy system (bioMerieux SA, Marct l’Etoile, France); HPV genotypes divided into HPV 16, HPV 18, and non-HPV 16/18 hrHPV.	no data	This study aimed to determine the prevalence of CIN2+ in women with persistent HPV, negative cytology, and TZ3; to stratify the risk of CIN2+; and to identify the best diagnostic strategy for women with TZ3.	multicenter retrospective cohort study; enrolled women with negative cytology and TZ3 among the 213 women referred for colposcopy for persistent HPV. Women with TZ3 underwent diagnostic LEEP to ensure correct diagnoses	The study highlighted 19% of CIN2+ lesions, a higher frequency of non-HPV 16/18 genotypes (76.2%), and 50% of CIN2+ lesions being due to non-HPV 16/18 genotypes. 80.9% of women had normal histopathological results in the LEEP sample. Women with viral persistence, negative cytology, and TZ3 have a 19% risk of CIN2+.	Genotyping helps stratify risk, but extensive genotyping is necessary instead of partial genotyping (16/18), referring to a population of women over 50 years old in which the prevalence of genotypes 16,18 decreases. Diagnostic LEEP is excessive, even though 83% of women had viral clearance after LEEP; p16/Ki67 double staining could be a potential risk marker, which would only highlight women at risk of CIN2+ to undergo LEEP	Persistent infection has been defined as the detection of the same type of HPV at ≥2 consecutive visits spaced ≥6 or ≥12 months apart. Incident or transient HPV infections are defined as the detection of a new HPV genotype.
Li M. et al. [34]	JH-DNA Methylation-Lightning MagPrep kit (OriginPoly Bio-Tec Co., Ltd., Beijing, China); the levels of PAX1 methylation (PAX1m) and JAM3 methylation (JAM3m), HPV genotyping and viral load were tested using BioPerfectus Multiplex Real-Time PCR	March 2023–December 2023	This study aims to investigate the correlation between the duration of persistent HPV infection and methylation as well as HPV VL, providing objective evidence for the monitoring and subsequent intervention of persistent HPV infection.	Females diagnosed with persistent HPV infection and pathologically confirmed lack of HSIL were categorized into two groups based on the duration of HPV infection: the HPV persistent less than 3 years group and the more than 3 years group. PAX1/JAM3 methylation and HPV VL were determined by real-time PCR and Real-Time (BMRT)-HPV reports type-specific VL/10,000 cells.	The methylation levels of JAM3 and PAX1 were significantly higher in individuals with HPV infection persisting for more than 3 years compared to those with less than 3 years, with a statistically significant difference (*p* < 0.05). There was a significant correlation between PAX1 and JAM3 methylation (*p* < 0.001). The incidence of vaginal intraepithelial lesions was higher in individuals with HPV infection persisting for more than 3 years compared to those with less than 3 years.	PAX1 and JAM3 methylation (*p* < 0.001) could be used as cumulative evidence of HPV infection duration before the occurrence of precancerous lesions. HPV VL can be used as an indicative biomarker for concurrent cervical–vaginal lesions, especially for HPV other than 16/18 genotypes.	Persistent HPV infection was defined as the persistent infection with the same HPV genotype for 1 year or more
Rezhake, R. et al. [35]	The careME methylation test (careLYFE, Suzhou, China) was used, which is based on methylation-specific real-time PCR techniques and targets the host cell gene EPB41L3 and viral HPV16L1/HPV18L2 genes	2018	To evaluate the cross-sectional and longitudinal performance (over 24 months) of methylation biomarkers targeting the human EPB4IL3 gene and the HPV 16/18 L1/L2 genes among hrHPV-positive women in comparison with cytology and genotyping	Three cervical exfoliated cell samples were collected from them for careHPV, PCR HPV, p16(INK4a), and liquid-based cytology (LBC) tests. Women with any positive test were referred for colposcopy, with biopsies taken at abnormal sites. Histopathological diagnoses were used as the gold standard.	The specificity (91.2%) of methylation was significantly higher than that of other triage methods (*p* < 0.001 for all). The longitudinal sensitivity of methylation over 24M follow-up was 56.0%, lower than HPV16/18 (64.0%) and cytology (76.0%). Methylation testing showed high PPV for CIN2+ (41.4% at baseline, 50.0% at 24 months), while the CIN2+ risk of methylation-negative women (cNPV) remained considerable (2.5% at baseline, 6.9% at 24 months).	Methylation has better specificity and predictive values for the presence or development of cervical precancer. It might therefore be considered for the strategy of HPV screening and methylation triage followed by immediate treatment of triage-positive women and delayed follow-up of hrHPV-positive/methylation-negative women.	no data
Bruno, M.T. et al. [36]	PreTect HPV-Proofer real-time multiplex NASBA test, using a primer/probe PCR for HPV types 16, 18, 31, 33, and 45	January 2012–December 2018	The goal was to develop an efficient method to identify patients who require a second LEEP from those who require FU using an mRNA-detection test.	In a population of 686 women undergoing a LEEP excision for CIN 3, we selected 285 women at risk of residual disease and subjected them to a search for E6/E7 mRNA HPV. The women with negative mRNA underwent follow-up, while those with positive mRNA underwent a second LEEP.	The histological examination of the second cone revealed 120 (85.7%) cases of residual disease in the mRNA-positive women: 40 cases of CIN2, 51 cases of CIN3, 11 cases of squamous microinvasive carcinoma, 7 cases of squamous carcinoma, 9 cases of AIS (adenocarcinoma in situ), and 2 cases of adenocarcinoma. Among the mRNA-negative women undergoing follow-up, only 5 cases of residual disease were identified. During the follow-up period of about 6 years, we observed regression of the residual disease and elimination of the virus, as predicted by the negative mRNA test result.	Testing patients for E6/E7 mRNA allowed us to identify women with residual disease (CIN2+) and treat them appropriately.	Residual disease is the diagnosis of CIN2+ at the first post-conization assessment; low-grade cervical lesions (LSIL/CIN1) were not considered as residual disease. The identification of the same type of HPV before and after the LEEP was considered HPV persistence.
Louvanto K. et al. [37]	QIAamp DNA Mini kit (Qiagen Inc, Hilden, Germany), DNA methylation of EPB41L3 and the late (L1 and/or L2) regions of HPV16, HPV18, HPV31, and HPV33	2013–2017	To investigate the ability of a DNA methylation panel (the S5 classifier) to discriminate between outcomes among young women with untreated CIN grade 2 (CIN2).	Baseline pyrosequencing methylation and human papillomavirus (HPV) genotyping assays were performed on cervical cells from 149 women with CIN2 in a 2-year cohort study of active surveillance.	The S5 classifier alone or in combination with HPV16/18/31/33 genotyping also showed significantly increased sensitivity vs. cytology when comparing regression vs. persistence/progression. With both the S5 classifier and cytology set at a specificity of 38.6% (95% confidence interval [CI], 28.4–49.6), the sensitivity of the S5 classifier was significantly higher (83.6%; 95% CI, 71.9–91.8) than cytology (62.3%; 95% CI, 49.0–74.4; *p* = 0.005).	The S5 classifier shows high potential as a prognostic biomarker to identify progressive CIN2.	no data
Lee H. et al. [38]	EZ DNA Methylation-Gold Kit (Zymo Research, Irvine, CA, USA)	January 2005–June 2013	The objective of the current study was to identify predictors of progression to HSIL and determine what percentage of ASC-US/LSIL cases harbor cervical intraepithelial neoplasia of grade 2 or higher.	165 cases with follow-up cytology were used to analyze predictive factors of progression to HSIL, and 129 cases that underwent immediate tissue biopsy were subjected to correlation analysis between cytology and histology.	Human papillomavirus infection was more common in women with disease progression than in those with disease regression or persistence (*p* = 0.033). Promoter methylation of p16 in cytology samples was more common in cases that progressed (5 of 6 cases) than in those that regressed (0 of 8 cases). Twenty-three of 129 cases (17.8%) were found to harbor cervical intraepithelial neoplasia of grade 2 or higher on immediate tissue biopsy.	Human papillomavirus infection and p16 promoter methylation may serve as valuable surrogate markers of disease progression from ASC-US/LSIL to HSIL.	Disease regression was defined as a reversion to normal or benign cellular changes, disease persistence as maintenance at ASC-US/LSIL, and disease progression as progression to HSIL.
Zhou Y. et al. [39]	The fluorescence-based multiplex HPV DNA genotyping kit (Bioperfectus Ltd., Taizhou, China). For the 21 most common HPV types, including 14 HR-HPV genotypes (HPV16, 18, 31, 33, 35, 39, 45, 51, 52, 56, 58, 59, 66, and 68) and 7 MR and LR-HPV genotypes (HPV26, 53, 82, 73, 6, 11, and 81).	September 2022–August 2023	This prospective study aims to examine the impact of changes in viral load on the occurrence of cervical lesions and to evaluate viral load as a biomarker for predicting cervical lesions and triaging HPV-positive patients.	All participants tested positive for HPV and negative for both cytology and pathology. A follow-up was conducted 6 months later to reassess HPV status and perform colposcopy.	At baseline, the viral loads of the virus clearance, maintenance, and progression groups demonstrated an increasing trend (*p* < 0.001). Among women diagnosed with CIN during follow-up, viral load increased significantly from baseline (*p* = 0.001).	It can be concluded that high viral load persistent infection is related to cervical lesions. HPV genotype and viral load may be able to predict the progression of cervical lesions. The application of HPV viral load to the first screening of cervical cancer can better predict and identify cervical lesions.	no data
Peronace, C. et al. [40]	A Qiasure (Qiagen) was used for FAM19A4 and hsa-miR124-2 methylation testing on bisulfite-converted DNA	2023	The study aimed to evaluate FAM19A4 and hsa-miR124-2 methylation in Atypical Squamous cells with high-grade squamous intraepithelial lesions (ASC-H) and in CIN1, defined as low-grade squamous intraepithelial lesions (LSILs) by the Bethesda classification, as possible early warning biomarkers for managing women with high-risk HPV infections (hrHPV).	FAM19A4 and hsa-miR124-2 methylation tests were conducted on fifty-six cervical screening samples from a subset of women aged 30–64 years old. Specimens were collected into ThinPrep PreservCyt Solution. Their HrHPV genotype and cytology diagnosis were known. A Qiasure (Qiagen) was used for FAM19A4 and hsa-miR124-2 methylation testing on bisulfite-converted DNA, according to the manufacturer’s specifications. The reported results were hypermethylation-positive or -negative.	FAM194A4 and hsa-miR124-2 methylation were detected in 75% of ASC-H cases with a persistent infection of hrHPV. A total of 60% of CIN1 lesions were found to be methylation-positive, and 83.3% were when cytology was CIN2/3. We found that combined FAM19A4 and hsa-miR124-2 methylation positivity rates (both methylated) were associated with HPV genotypes 16, 18, and 59 and accounted for 22% and 25% of ASC-H and CIN1 cases, respectively.	The methylation of these two genes, in combination with HPV genotyping, can be used as an early warning biomarker in the management and follow-up of women with ASC-H and CIN1 to avoid their progression to cervical cancer.	no data
Fertey J. et al. [41]	INNO-LiPA HPV Genotyping v2 test; 454 sequencing was carried out on the Titanium platform (Roche/454 Life Sciences) by Eurofins/MWG Operon, Erlangen, Germany; The consensus sequence was generated with MegAlign (SeqMan NGen^®^)	1995–2014	This study aimed to investigate whether methylation at a specific site could predict the development of viral persistence and whether viral load correlates with specific methylation patterns.	HPV16-positive samples from women aged 20–29 years (*n* = 99) with a follow-up time of 13 years were included from a Danish cohort comprising 11,088 women. Viral load was measured by real-time PCR, and methylation status was determined for 39 CpG sites in the upstream regulatory region (URR), the E6/E7 region, and the L1 region of HPV16 by next-generation sequencing.	The general methylation status was significantly different between women with a persistent and women with a transient infection outcome (*p* = 0.025). One site located in L1 (nt. 5962) was statistically significantly (*p* = 0.00048) different in the methylation status after correction using the Holm–Sidak method (alpha = 0.05).	Correlation analyses of samples from HPV16-persistently infected women suggest that methylation is higher, even though viral load is lower. This study indicates that methylation at position 5962 of the HPV16 genome within the L1 gene might be a predictive marker for the development of a persistent HPV16 infection.	
Pruski D. et al. [42]	The DNA methylation detection kit for human *PAX1*, *SOX1*, and *HAS1* is 1% for each gene. Real-time PCR was performed using a Gentier96R Real-time PCR System (Xian Tianlong Science and Technology Co., Ltd., Shanglin Road, Weiyang District, Xian, Shaanxi, China).	2022–2023	To avoid the overtreatment of low-grade squamous intraepithelial lesions (LSILs), especially in young women, methylation testing may be considered.	In patients with histopathologically confirmed high-grade squamous intraepithelial lesions (HSILs) during biopsy and, ultimately, a lower diagnosis (i.e., LSIL or no signs of atypia), methylation was found to be a useful tool. We performed a Pap smear, HPV genotyping, a punch biopsy, LEEP-conization (if needed), and methylation tests on 108 women	Women with a negative methylation test result were significantly more likely to be ultimately diagnosed with LSIL (*p* = 0.013). This means that in 85.7% of the patients with HSIL, major cervical surgery could be avoided if the methylation test were negative.	The methylation test may also be used in the diagnosis and identification of lesions within the cervical canal, including those located deep within the frontal crypts, which are not visible even during a professional colposcopic evaluation of the cervix.	no data

## Data Availability

No new data were created or analyzed in this study. Data sharing is not applicable to this article.
