# Correction: Pruski et al. Analysis of Molecular Markers of HPV Infection Persistence: A Narrative Review. *Cancers* 2026, *18*, 981

**DOI:** 10.3390/cancers18152441

**Published:** 2026-07-29

**Authors:** Dominik Pruski, Sonja Millert-Kalińska, Katarzyna Wszołek, Victoria Musiałowicz, Jacek P. Grabowski, Robert Jach, Mustafa Zelal Muallem, Jalid Sehouli, Marcin Przybylski

**Affiliations:** 1Department of Obstetrics and Gynecology, District Public Hospital in Poznan, 60-479 Poznan, Poland; millertsonja@gmail.com (S.M.-K.);; 2Department of Maternal and Child Health, Poznan University of Medical Sciences, 60-806 Poznan, Poland; 3Department of Gynecology with Center for Oncological Surgery, Charité-University Medicine of Berlin, Campus Virchow Klinikum, 13353 Berlin, Germanymustafa-zelal.muallem@charite.de (M.Z.M.);; 4Division of Gynecologic Endocrinology, Jagiellonian University Medical College, 31-501 Krakow, Poland

## Text Correction

In the original publication [1], there was a mistake in two texts of Section 3.2.

Original text 1:

“Currently, there are three most common commercial tests for detecting hrHPV E6/E7 messenger RNA (mRNA). The PreTect HPV-Proofer (NorChip AS, Klokkarstua, Norway) and the NucliSENS Easy Q HPV (bioMérieux, Marcy-l’Étoile, France) are based on the same technology. Still, they are produced by different companies, with small differences in mRNA extraction protocol and data analysis [22,23].”

Original text 2:

“The advantage of using HPV mRNA tests is their similar sensitivity combined with slightly higher specificity compared to HPV DNA tests for the detection of CIN 2+ lesions [25]. Furthermore, HPV mRNA tests can also be used for follow-up after treatment for CIN lesions (test-of-cure). Limitations of this method include a limited number of detectable genotypes and the fact that they cannot be used combined with self-sampling for cervical cancer screening.”

A correction has been made to Section 3.2. HPV mRNA Test, the corrected texts are as follows:

Corrected text 1:

“PreTect AS currently markets both a 3-type assay (PreTect SEE), the 5-type assay PreTect HPV-Proofer and a 7-type assay (PreTect HPV-Proofer 7), whereas NucliSENS Easy Q HPV was historically marketed by bioMérieux under license from NorChip technology and is no longer manufactured. The PreTect HPV-Proofer (NorChip AS, Klokkarstua, Norway) and the NucliSENS Easy Q HPV (bioMérieux) differs slightly in mRNA extraction protocol and data analysis [22,23].”

Corrected text 2:

“The advantage of some HPV mRNA tests, particularly genotype-specific assays used in triage, is their comparable sensitivity combined with higher specificity than HPV DNA testing for the detection of CIN2+ lesions [25]. Furthermore, HPV mRNA assays may be used for follow-up after treatment for CIN lesions (test-of-cure) and can also be combined with self-sampling in appropriate settings [20]. Some HPV mRNA assays target selected high-risk genotypes rather than the full spectrum of hrHPV types; however, in triage this selective genotype coverage may also represent a strength by improving specificity and risk stratification.”

The authors state that the scientific conclusions are unaffected. This correction was approved by the Academic Editor. The original publication has also been updated.

## References

In the published article [1], Reference 23, originally cited as Muenchhoff et al., has been corrected to the intended reference, Munkhdelger et al. The corresponding in-text citation number remains unchanged. The authors state that the scientific conclusions are unaffected. This correction was approved by the Academic Editor. The original publication has also been updated.

Original ref. [23]:

23. Muenchhoff, M.; Madurai, S.; Hempenstall, A.J.; 
Adland, E.; Carlqvist, A.; Moonsamy, A.; Jaggernath, 
M.; Mlotshwa, B.; Siboto, E.; Ndung’U, T.; et al. 
Evaluation of the NucliSens EasyQ v2.0 Assay in 
Comparison with the Roche Amplicor v1.5 and the Roche 
CAP/CTM HIV-1 Test v2.0 in Quantification of C-Clade 
HIV-1 in Plasma. *PLoS ONE* **2014**, *9*, e103983. 
https://doi.org/10.1371/journal.pone.0103983.

Corrected ref. [23]:

23. Munkhdelger, J.; Choi, Y.; Lee, D.; Kim, S.; Kim, G.; Park, S.; Choi, E.; Jin, H.; Jeon, B.Y.; Lee, H.; et al. Comparison of the performance of the NucliSENS Easy Q HPV E6/E7 mRNA assay and HPV DNA chip for testing squamous cell lesions of the uterine cervix. *Diagn. Microbiol. Infect. Dis.* **2014**, *7*, 422–427. https://doi.org/10.1016/j.diagmicrobio.2014.04.004.
